# Transcriptomic analysis of cultivated cotton *Gossypium hirsutum* provides insights into host responses upon whitefly-mediated transmission of cotton leaf curl disease

**DOI:** 10.1371/journal.pone.0210011

**Published:** 2019-02-07

**Authors:** Rubab Zahra Naqvi, Syed Shan-e-Ali Zaidi, M. Shahid Mukhtar, Imran Amin, Bharat Mishra, Susan Strickler, Lukas A. Mueller, Muhammad Asif, Shahid Mansoor

**Affiliations:** 1 Agricultural Biotechnology Division, National Institute for Biotechnology and Genetic Engineering (NIBGE), Faisalabad, Punjab, Pakistan; 2 Pakistan Institute of Engineering & Applied Sciences (PIEAS), Nilore, Islamabad, Pakistan; 3 Boyce Thompson Institute, Cornell University, Ithaca, NY, United States of America; 4 Department of Biology, University of Alabama at Birmingham, Birmingham, AL, United States of America; National Institute of Plant Genome Research, INDIA

## Abstract

Cotton is a commercial and economically important crop that generates billions of dollars in annual revenue worldwide. However, cotton yield is affected by a sap-sucking insect *Bemisia tabaci* (whitefly), and whitefly-borne cotton leaf curl disease (CLCuD). The causative agent of devastating CLCuD is led by the viruses belonging to the genus *Begomovirus* (family *Geminiviridae*), collectively called cotton leaf curl viruses. Unfortunately, the extensively cultivated cotton (*Gossypium hirsutum*) species are highly susceptible and vulnerable to CLCuD. Yet, the concomitant influence of whitefly and CLCuD on the susceptible *G*. *hirsutum* transcriptome has not been interpreted. In the present study we have employed an RNA Sequencing (RNA-Seq) transcriptomics approach to explore the differential gene expression in susceptible *G*. *hirsutum* variety upon infection with viruliferous whiteflies. Comparative RNA-Seq of control and CLCuD infected plants was done using Illumina HiSeq 2500. This study yielded 468 differentially expressed genes (DEGs). Among them, we identified 220 up and 248 downregulated DEGs involved in disease responses and pathogen defense. We selected ten genes for downstream RT-qPCR analyses on two cultivars, Karishma and MNH 786 that are susceptible to CLCuD. We observed a similar expression pattern of these genes in both susceptible cultivars that was also consistent with our transcriptome data further implying a wider application of our global transcription study on host susceptibility to CLCuD. We next performed weighted gene co-expression network analysis that revealed six modules. This analysis also identified highly co-expressed genes as well as 55 hub genes that co-express with ≥ 50 genes. Intriguingly, most of these hub genes are shown to be downregulated and enriched in cellular processes. Under-expression of such highly co-expressed genes suggests their roles in favoring the virus and enhancing plant susceptibility to CLCuD. We also discuss the potential mechanisms governing the establishment of disease susceptibility. Overall, our study provides a comprehensive differential gene expression analysis of *G*. *hirsutum* under whitefly-mediated CLCuD infection. This vital study will advance the understanding of simultaneous effect of whitefly and virus on their host and aid in identifying important *G*. *hirsutum* genes which intricate in its susceptibility to CLCuD.

## Introduction

Cotton (*Gossypium hirsutum L*.*)* is one of the most important economic crops grown on several continents of the world. It is cultivated in more than eighty countries around the world, including China, India, USA and Pakistan [1]. It is a principal source of high quality fiber that makes cotton the backbone of the textile industry. Cotton is also important owing to its high-quality protein and oil rich seed that is used for cooking oil production and livestock feed [2]. However, several biotic and abiotic stresses affect the overall cotton production and fiber quality. The annual crop loss in cotton due to biotic stresses is significantly higher than any other agriculturally important crops.

The major devastating biotic factors that significantly reduce cotton quality and production include insect pests that accounts for up to about 37% yield losses [3]. *Bemisia tabaci*, or whitefly, is among the most devastating sap-sucking pest that directly causes more than 50% of the crop loss [4]. Additionally, it aids viruses as vector to transmit many bipartite begomoviruses like Cotton leaf curl crumple virus (CLCrV) and Cotton leaf curl virus (CLCuV) [5, 6]. CLCuV causes CLCuD in host plant [7] that is also responsible for serious crop losses annually [8]. Whitefly transmits a complex of single-stranded DNA viruses belonging to the genus *Begomovirus* (family *Geminiviridae*) along with their associated DNA satellites to establish CLCuD (alphasatellite and betasatellite) [9–12]. The CLCuD is manifested by the characteristic symptoms of upward or downward leaf curling, vein swelling, leaf enation, and growth stunting. The strategies adopted to improve insect or disease resistance traits include breeding approaches, RNAi, protein-mediated resistance, genetic engineering or genome editing techniques [11, 13, 14]. However, these viruses may develop resistance by mutations or recombination [15]. Therefore, it is essential to decipher the host responses during CLCuD to effectively control this deadly disease in cotton.

Plants have evolved a wide variety of intrinsic defense mechanisms to fight off pathogens including pests and viruses [16]. Upon a pathogen attack, plants undergo a dramatic transcriptional reprogramming by differentially expressing a vast array of genes leading to global changes in a variety of physiological and metabolic processes [17, 18]. On contrary, viruses are evolving by adapting to their hosts *via* evading host defense mechanisms and taking control of the host cellular machinery for their own benefits. Changes in the cellular metabolism along with differential gene expression as host antiviral responses contribute to virulence and establishment of viral disease [19].

Next-generation based RNA sequencing (RNA-seq) has emerged as a powerful tool to detect differentially expressed genes (DEGs) in several plant species [20]. Identification of such DEGs under a particular stress allows to understand the mechanisms of the complex nature of plant-microbe interactions [21] and engineering of broad-spectrum disease resistance [22]. At present, the available cotton transcriptome data is focused on salt/drought stress, fiber biology, leaf senescence, aphid resistance, verticillium resistance, whitefly resistance but not on CLCuD response [23–27]. Thus, we aim to study the response of tetraploid susceptible cotton *G*. *hirsutum* at a molecular level in response to whitefly-mediated CLCuD by using RNA-Seq technology. This study will help us in identification of disease-related DEGs, which can be further exploited to understand the cotton-whitefly-CLCuD interaction for effective control of disease.

## Materials and methods

### Whitefly-mediated CLCuD inoculation of cotton plants

Seeds of CLCuD susceptible cotton variety “Karishma” were obtained from Nuclear Institute of Agriculture and Biology, Faisalabad. Plants of *G*. *hirsutum*, CLCuD susceptible genotype “Karishma” were grown in a whitefly-free glasshouse. Three-week-old plants were then divided into two sets. One set of plant was maintained in whitefly-free conditions, while the other set was allowed to grow under viruliferous whiteflies (containing CLCuV/CLCuMB_Bur_) infestation. Each set contained 20 plants and we referred set one as a control and set two as infected. Glasshouse temperature was retained 38–45°C and 25–30°C for day and night respectively. After 25 days post infestation, when disease symptoms were very clear on the CLCuD infected plants, leaf tissues were taken from both CLCuD infected and control plants.

### RNA isolation and RNA-Seq library preparation

Leaf samples from three biological replicates of control and infected plants were subjected to total RNA isolation via Trizole method (Plant RNA reagent Invitrogen, USA). RNA quantity and quality was measured using NanoDrop 1000 spectrophotometer and 1% agarose gel electrophoresis respectively. Moreover, Bio-analyzer 2100 equipment was used to examine the RNA integrity. cDNA was synthesized using 10 μg of total RNA and all six samples were subjected to construction of strand specific libraries for RNA-Seq [28]. Oligo dT beads were used to extract poly (A) mRNA from the total RNA. The extracted mRNA was sheared into small fragments of about 300 nucleotides. Afterwards first and second strand cDNA were made with random hexamer-primers, RNaseH and DNA polymerase I. Purification and washing of cDNA fragments was done to end repair, followed by their ligations to adapters for sequencing. Finally, the cDNA libraries were attained after purification and PCR enrichment. cDNA libraries were inspected for integrity using Bio-analyzer 2100.

### Sequencing and transcriptomic data analysis

All libraries were pooled to be sequenced by HiSeq 2500 on single-end mode. All the low quality reads including adopter sequences were removed using Trimmomatic software [29]. Quality of untrimmed and trimmed reads was examined by FastQC [30]. HISAT2 aligner was used to map the clean reads to the *G*. *hirsutum* reference genome [31]. HISAT2-bulid was used to index the reference genome and HISAT2-align was run with default parameters allowing up to 2 mismatches and twenty alignments report. HISAT generated alignment was subjected to cuffdiff package of Cufflinks software for determining differential gene expression [32]. The statistical model of Cufflinks-cuffdiff measures the differential gene expression by using a unit FPKM *i*.*e*. Fragments per Kilobase of exon model per Million mapped reads. Cuffdiff was set to calculate abundance of differentially expressed genes (DEGs) between control and infected samples by using a cutoff q value of 0.05. An R-based Heatmap 2.0 package was implemented to make a hierarchical clustering heat map on Log2 expression values of the *G*. *hirsutum* differentially expressed genes under whitefly-mediated CLCuD infestation.

### RT-qPCR analysis for validation of RNA-Seq data

We performed a quantitative real-time RT-PCR (RT-qPCR) on independent samples of control and infected Karishma plants to substantiate the differential expression identified by RNA sequencing data. Primer3 program was used to design primers on 10 selected genes. Karishma leaves from control and infected samples were collected and flash frozen in liquid nitrogen. Total RNA was isolated from frozen samples using Trizol method and then subjected to DNase treatment to remove any DNA contamination. Two μg of total RNA was used for cDNA synthesis using Superscript II reverse transcriptase (Thermo-Fischer Scientific) as per manufacturer instructions. cDNA was diluted 5 times and 2.5 μL /reaction was used as a template. Expression analysis was performed using RT-qPCR using iQ5 Real-Time Bio-Rad PCR instrument. Each reaction was performed in 20 μL total volume with 10 μL SYBR Green Master Mix (Thermo-Fischer Scientific), 2.5 μL of cDNA template, 0.5 μL from 10 μM/μL of each primer and remaining volume was made up using nuclease free water. The amplification program was set using 95°C for 5 min of initial denaturation, followed by 35 cycles of 95°C for 15 s,and 55°C for 20 s. At the end, melt curve analysis was done at temperature range from 55 to 95°C to make sure the product specificity. ΔΔ Ct method was used to find out the relative gene expression taking 18s rRNA internal control gene for data normalization. To evaluate the implication of RNA-Seq data on other CLCuD susceptible cultivars, we did an additional RT-qPCR on samples of another CLCuD susceptible cultivar of *G*. *hirsutum* “MNH786”. RT-qPCR was performed on control and infected samples using same procedure as described above.

### WGCNA and network analyses

Construction of weighted gene-based co-expression network (WGCNA) was done using FPKMs of all 468 DEGs found in RNA-Seq data. An R-based package was run for WGCNA on FPKMs of DEGs [33–35]. CutreeDynamicTree algorithm was implemented with smallest module of 70 genes size as a set threshold to construct the dendrogram [33]. Different modules presented the entire network with respective colors were identified with a set weighted correlation cutoff ≥ 0.85. NetworkX Plugin was used to analyze degree of the network while CytoscapeV.3.5.1 was utilized for cluster coefficient analysis [36, 37]. “Group Attributes Layout”, an attribute of CytoscapeV.3.5.1 Plugins was employed for visualizing the co-expression network [38, 39].

### Functional annotation and GO-term analysis of DEGs

All DEGs were subjected to be searched in Cottongen database for the assignment of their respective biological functions. All these genes were also analyzed using Kobas 3.0 [40] for determination of associated GO-terms. An interactive graph of GO-terms with a threshold of 0.05 p-value was made by AgriGO toolkit [41]. Additionally, the identified modules and hub genes by WGCNA were also used for GO-term analysis to examine their probable role in cotton response to whitefly-mediated CLCuD.

### Data accessibility

RNA Sequencing raw data of present study is available at the National Center for Biotechnology Information (NCBI) as BioProject accession No. PRJNA398803.

## Results and discussion

Transcriptomic data on geminivirus-infected plants are very limited. A few microarray studies dealing with the impact of geminivirus infection on host gene expression are done with a focus on the model plant *Arabidopsis thaliana* [42, 43]. Using RNA-Seq, mostly studies have been done on geminivirus infected tomato or tobacco plants [44–46]. There are two reports that have studied transcriptome of cotton under aphid and/ whitefly infestation [26, 27]. Another recent RNA-Seq dataset has depicted the transcriptome changes in *G*. *arboreum* under graft-mediated CLCuD stress [47]. However, these studies are different from our study in terms of infestation, method of inoculation, cotton variety/genotype, ploidy level of cotton species and differential gene expression. Compared to previous studies, here we have presented a simultaneous effect of whitefly and CLCuV (field like condition) on cotton transcriptome. To our knowledge this is the first transcriptomic study in tetraploid cotton *G*. *hirsutum* under whitefly-mediated CLCuD infestation which provides comprehensive insights into the cotton responses to whitefly mediated CLCuD infestation.

### Cotton transcriptome reveals global transcriptional changes in response to CLCuD infection

**Fig 1:**
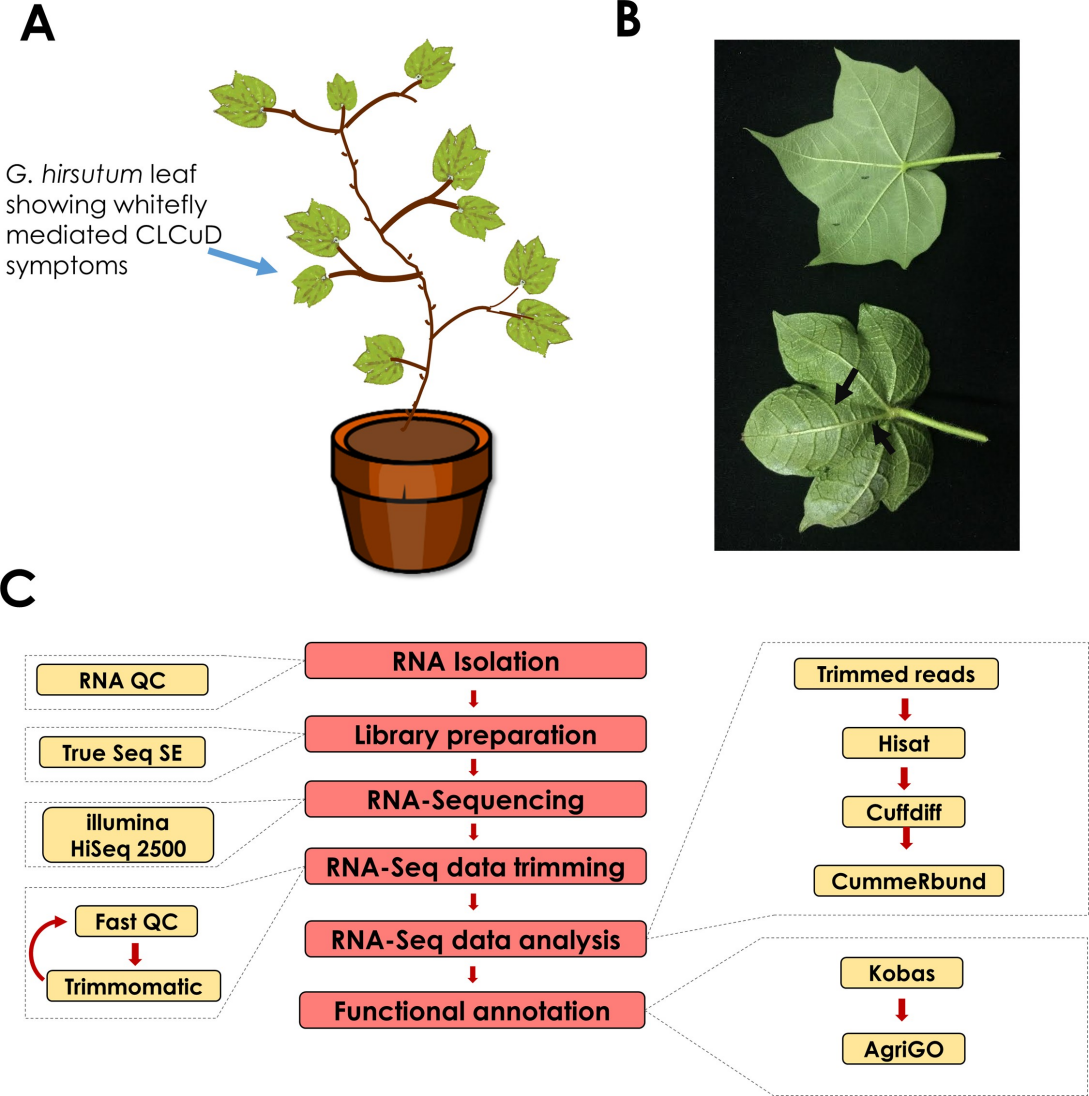
RNA-Seq of *G*. *hirsutum* under whitefly-mediated CLCuD stress. (A) Sketch of CLCuD infestation of three-week-old cotton plant with viruliferous whitefly. (B) Control (top) and CLCuD infected (bottom) leaf of *G*. *hirsutum* used for RNA-Seq, arrows are showing vein thickening in CLCuD infected leaf, (C) RNA-Seq data analysis methodology.

Notably, cultivated tetraploid cotton *G*. *hirsutum* is highly susceptible to CLCuD. Hence, the current study sheds light on the changes in gene expression of *G*. *hirsutum* in response to whitefly-mediated CLCuD. This study will also help decipher potential disease susceptibility mechanisms involved in *G*. *hirsutum* in response to the whitefly vector and begomoviruses causing CLCuD. A workflow for this transcriptomic study is highlighted in **Fig 1**.

**Fig 2:**
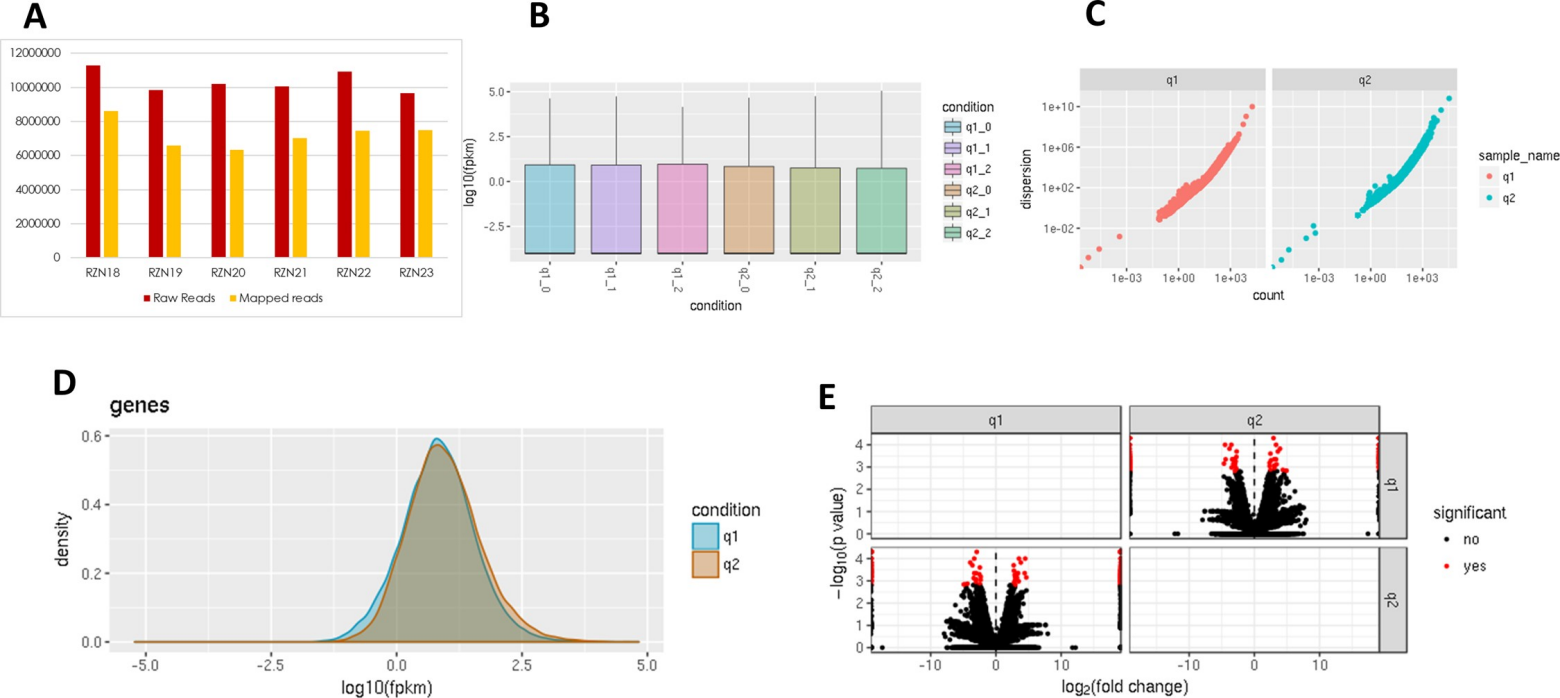
Data quality analysis and mapping to reference genome. (A) RNA-seq raw vs mapped reads where number of samples and reads lie on x and y-axis, respectively, (B) Data (Log10 FPKM) among the biological replicates, where q1 and q2 represent control and infected samples, moreover q1_0, q1_1, q1_2 symbolize three biological replicates of controls, q2_0, q2_1, q2_2 denote three biological replicates of infected samples. (C-E) Gene dispersion, density and DEGs in the dataset.

**Fig 3:**
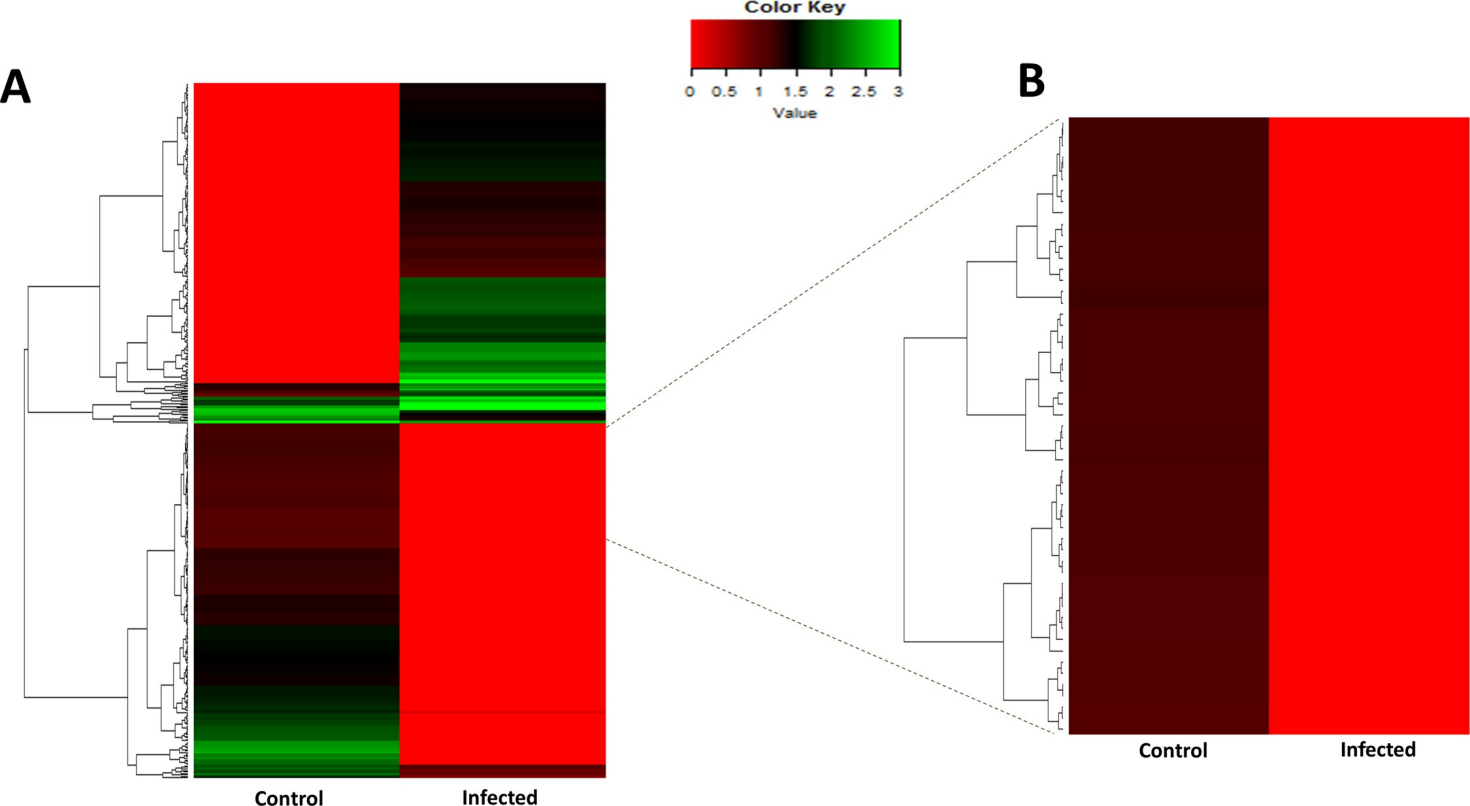
Hierarchical clustering heatmap of cotton DEGs under whitefly-mediated CLCuD. R-based Heatmap 2.0 package was implemented to make a heat map of *G*. *hirsutum* DEGs under whitefly-mediated CLCuD stress (A). Heatmap of 468 DEGs and (B). Hub genes identified by WGCNA.

High quality RNA was isolated from leaf tissues obtained from control and CLCuD infected plants in three biological replicates. After library preparation, RNA-Seq was performed on these pooled libraries using Illumina HiSeq 2500 platform. For each of the six samples, we obtained an average of 10 million reads per sample (**Fig 2A**). These reads were checked for quality by FastQC before and after Trimmomatic processing with a high base call Phred Score 64 (**S1 Fig**). The mapping of high quality control sample reads RZN18-20 and infected samples RZN22-24 was performed using HISAT2 to the reference genome of *G*. *hirsutum* (26 chromosomes and 76,943gene models), alignment summary is shown in **Fig 2A**. Subsequently, control and infected samples alignment was used to find out the differential gene expression. Biological replicates were compared individually for each condition and transcript abundance was calculated using Cuffdiff [32] (**Fig 2B**). As a quality check, gene density and genes dispersion was provided by comparison of transcripts (**Fig 2C and 2D**). FPKM value was used to normalize the mapped genes expression levels. Log2 fold change greater than 1 and a cutoff false discovery rate < 0.05 was customized to identify DEGs in comparison of control and disease infected leaves of *G*. *hirsutum* plants that yielded 468 DEGs as provided in **S1 Table**. Among them, 220 up and 248 genes were found downregulated (**Figs 2E and 3A**).

To corroborate our Karishma transcriptomic data, we selected 10 significantly DEGs having their potential roles in disease susceptibility (**Tables 1**and **S2**). These genes include CRD, COBL7, arginine decarboxylase, amino-acid decarboxylase and alpha-galactosidase 1 (AGAL-1), WEB1, CDF, 1-aminocyclopropane-1-carboxylate oxidase (ACO1) and Metallothionein-like protein. Differential expression of these genes suggest their involvement in CLCuD response in cotton. Moreover, the role of these genes, based on many previous reports has been discussed below.

**Table 1:** List of some significant differentially expressed genes in the transcriptomic data of *G*. *hirsutum* under whitefly-mediated CLCuD infection.

Gene_ID	Function	Control_FPKM	Infected_FPKM	Regulation in *G*. *hirsutum*
CotAD_04678	Metallothionein-like protein 1	0	1357.24	up
CotAD_19598	COBRA-like protein 7	218.176	0	down
CotAD_19166	Arginine decarboxylase	66.2789	7.13967	down
CotAD_30209	1-aminocyclopropane-1-carboxylate oxidase (ACO1)	21.9758	183.178	up
CotAD_28371	alpha-galactosidase 1	110.121	10.4329	down
CotAD_24161	Aromatic L. amino acid decarboxylase	256.185	0	down
CotAD_62004	WEB family protein	0	178.295	up
CotAD_28260	Magnesium-protoporphyrin IX monomethyl ester [oxidative] cyclase	363.941	32.3541	down
CotAD_53254	Magnesium-protoporphyrin IX monomethyl ester [oxidative] cyclase	491.584	34.9266	down
CotAD_10143	cyclic dof factor 3	11.0365	55.8462	up

The upregulation of chlorophyll biosynthesis-related two CRD genes and downregulation of WEB1, a chloroplast photodamage responsive gene was observed. It’s important to note that host genes involved in chloroplast biosynthesis and development were previously shown to interact with BC1 of a Radish leaf curl disease (RaLCB) in *Nicotiana benthamiana*. This interaction ultimately leads to a damage of chloroplast integrity for successful viral symptoms establishment [48, 49]. ACO1 that is induced in infected Karishma leaves is involved in ethylene formation and pathogen defense [50]. Previously, expression of ACO1 was shown to be elevated in plants because of severe necrosis caused by the with Potato Virus Y infection [51]. Besides metal binding, metallothionein proteins are involved in capturing of harmful oxidant radicals. Moreover, expression levels of metallothionein in plants is induced to combat oxidative stresses [52]. Hence, upregulation of metallothionein protein in our data represents its involvement in cell survival under oxidative stress caused by whitefly and CLCuD. Arginine decarboxylase catalyzes the arginine metabolism and therefore involved in polyamine synthesis. The disease resistance response of Polyamines is related to ROS that inhibit pathogen growth, stimulate cross-linking of the plant cell wall and mediate defense responsive pathways [53]. The downregulation of arginine decarboxylase in transcriptomic data here depicts its role in disease susceptibility. Amino-acid decarboxylase gene exhibit suppression under sap-sucking insects and viral stresses [54, 55], therefore, we found this gene to be downregulated by whitefly and CLCuD stress response. Upregulation of CDF3 denotes its probable role in disease stress response as observed previously in pepper upon infection with different viruses [56]. The downregulation of two secondary cell wall biosynthesis and biogenesis related genes, COBL and AGAL-1 denote their role in cell wall mediated signal transduction upon pathogen attack [57, 58].

**Fig 4:**
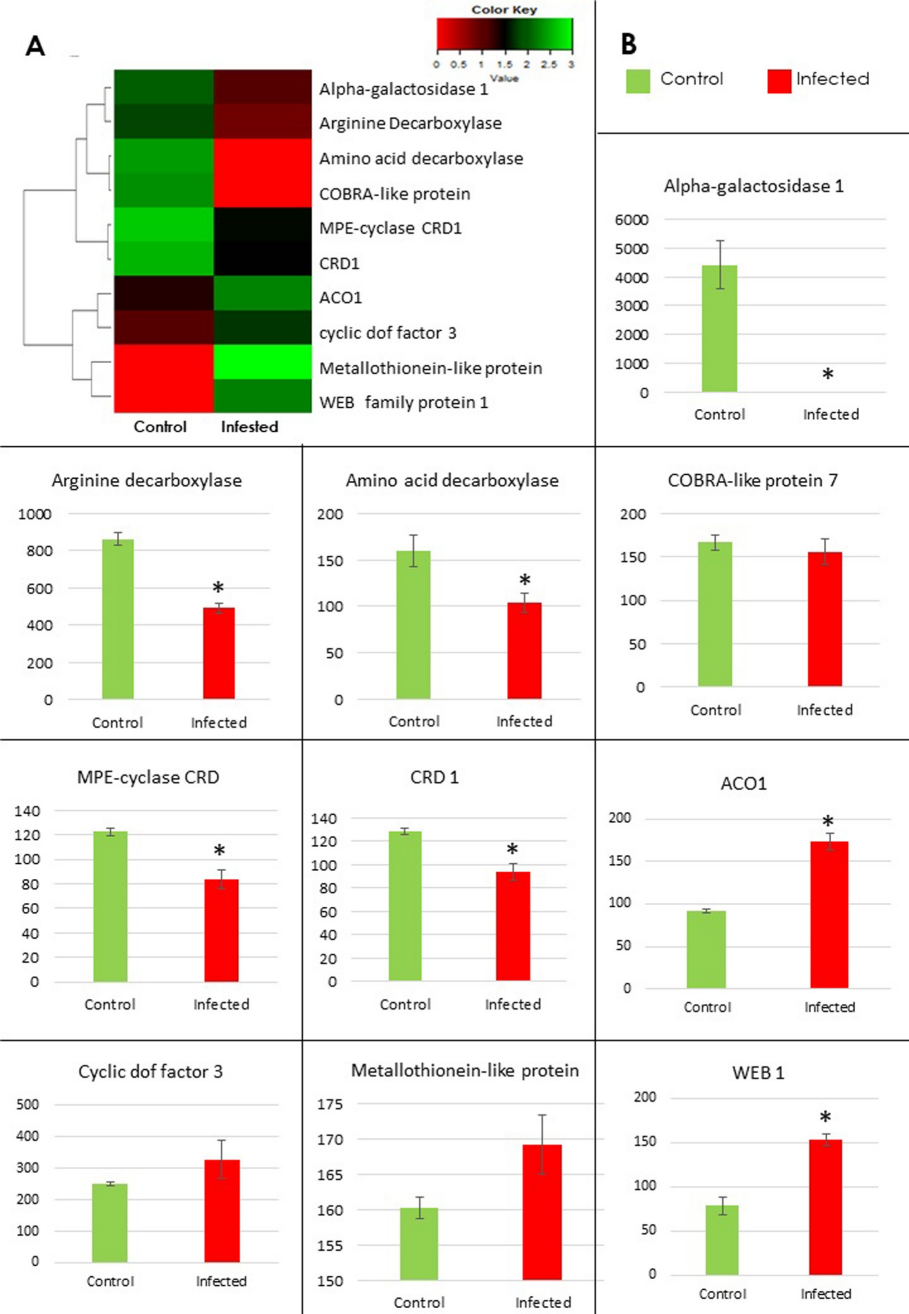
RT-qPCR Validation of transcriptomic data in Karishma cultivar. (A) R-based heat map of 10 selected DEGs for RT-qPCR. (B) Relative expression levels of selected ten genes and 18S as an internal reference detected by RT-qPCR in control and symptomatic leaves. Error bars symbolized standard error of three biological replicates and * shows significance determined by Student’s t-test.

**Fig 5:**
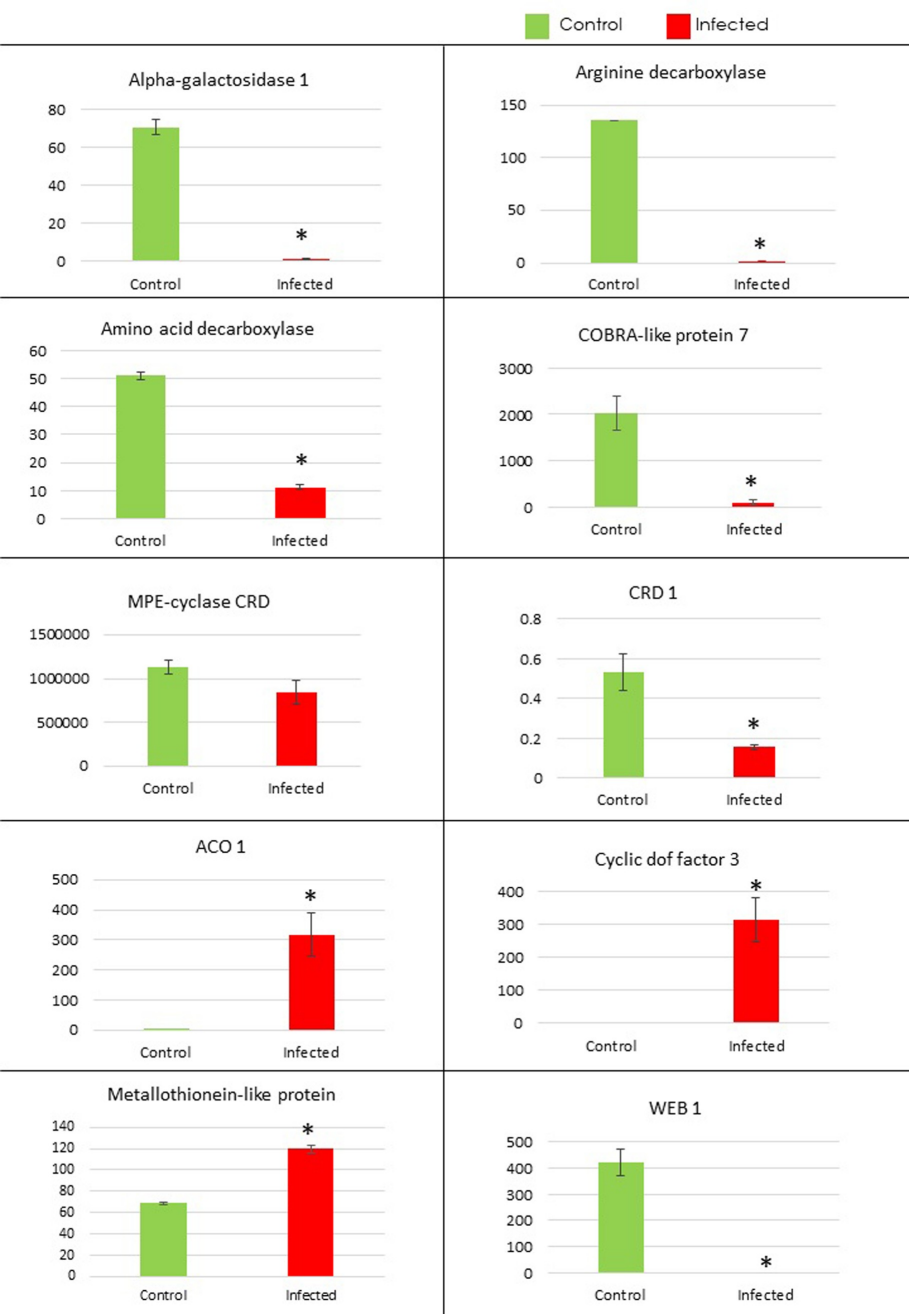
RT-qPCR Validation of transcriptomic data in MNH786 cultivar. Relative expression levels of selected ten genes and 18S as an internal reference detected by RT-qPCR in control and symptomatic leaves. Error bars symbolized standard error of three biological replicates and * shows significance determined by Student’s t-test.

We examined the transcriptional responses of these genes in two susceptible cultivars, Karishma and MNH786 using RT-qPCR analyses. Karishma has been found to be highly susceptible to CLCuD during field observations [59], therefore, we used this variety in transcriptomic study to decipher mechanisms impartially involved in disease susceptibility. However, MNH786 is relatively a recent cotton variety that is also susceptible to CLCuD [60], so we hypothesized that both susceptible cultivars might exhibit similar transcriptomic changes under CLCuD infestation. Our qPCR data on independent biological replications of highly susceptible Karishma corroborated with RNA-Seq expression (**Fig 4**). Intriguingly, transcript levels of all the tested genes in qPCR, with one exception displayed analogous expression patterns in MNH786, another susceptible *G*. *hirsutum* cultivar (**Fig 5**).

Perhaps, one gene that did not show consistent mRNA levels between two susceptible cultivars might be due to genotype dependent gene expression or different sensitivity of two techniques. Overall, our qPCR findings demonstrate the implication of our transcriptomic data on other CLCuD susceptible cultivars. To gain further insights into this transcriptome data, we performed gene set enrichment analysis. We discovered several functional sets of DEGs, significantly altered in CLCuD infected cotton plants particularly involved in pathogen interaction, defense and disease susceptibility.

### Functional annotation unveils differential gene expression of important gene families in cotton upon CLCuD infection

**Fig 6:**
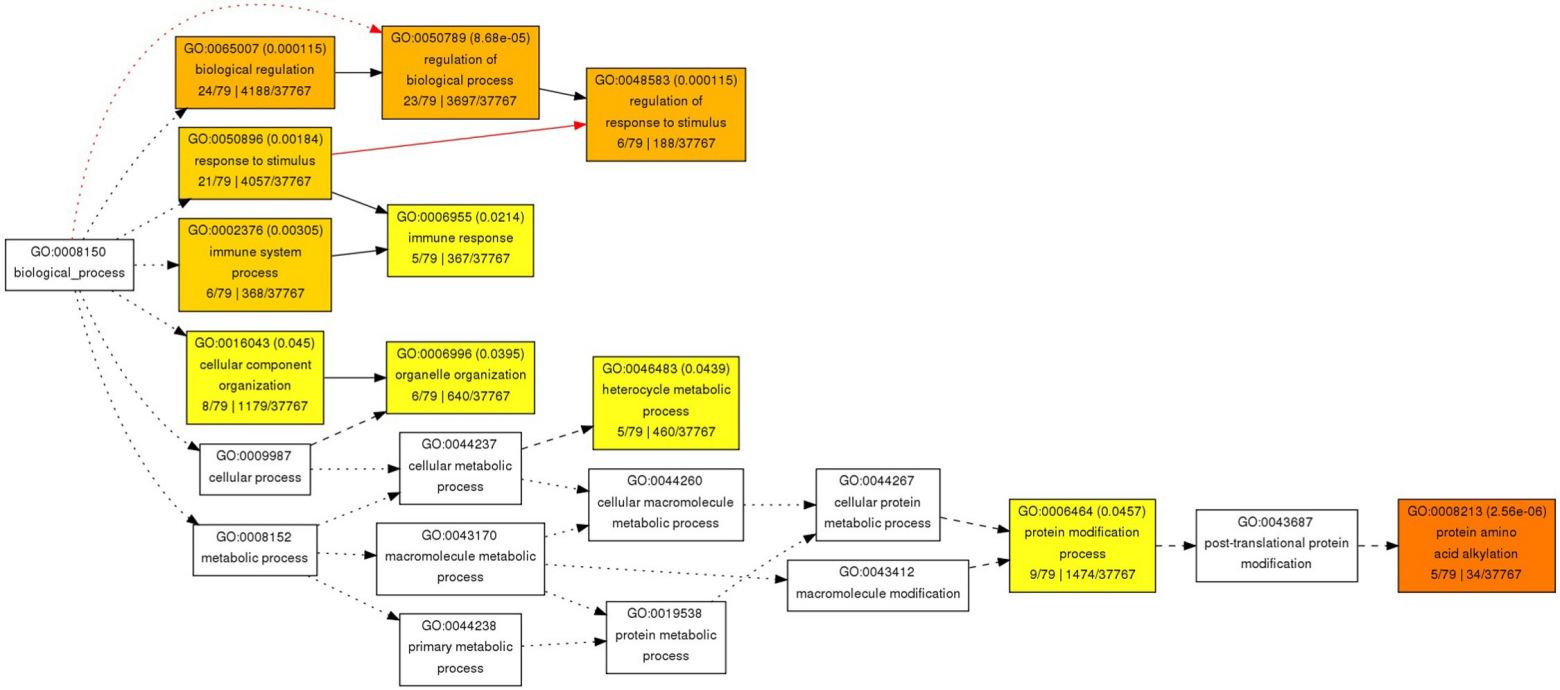
GO term analysis of DEGs. GO term annotation of *G*. *hirsutum* differentially expressed genes under CLCuD stress.

On the course of evolution, plants have established sophisticated defense mechanisms for coping with pests and pathogens including viruses and insects [61]. In addition to physical barriers (such as trichomes as well as toxic compounds and secondary metabolites), plants employ an intricate network of defense signaling pathway to defend themselves against a wide-range of pathogens [62]. On contrary, pests and pathogens like insects and viruses have evolved counter-defense strategies by hijacking host machinery to compromise host defenses and establish disease susceptibility [63]. This CLCuD-related transcriptome analysis sheds light on such mechanisms that suppress plant immune system, avert pathogen recognition and influence host metabolic pathways to establish disease susceptibility in cotton. Functional annotation using a GO enrichment analyses of DEGs in *G*. *hirsutum* plants under CLCuD, shown several groups of genes differentially expressed in cotton in our study. The majority of these genes include metabolite synthases, transcription factors, protein kinases and phytohormone-related genes. GO terms associated with DEGs were found to be related to biological processes, immune response, organelle organization, heterocyclic metabolism and protein modification-related processes (**Fig 6**). We discuss here the potential roles of these functional classes in the context of network structural analyses in the succeeding section.

### Co-expression gene network pinpoints six novel modules

**Fig 7:**
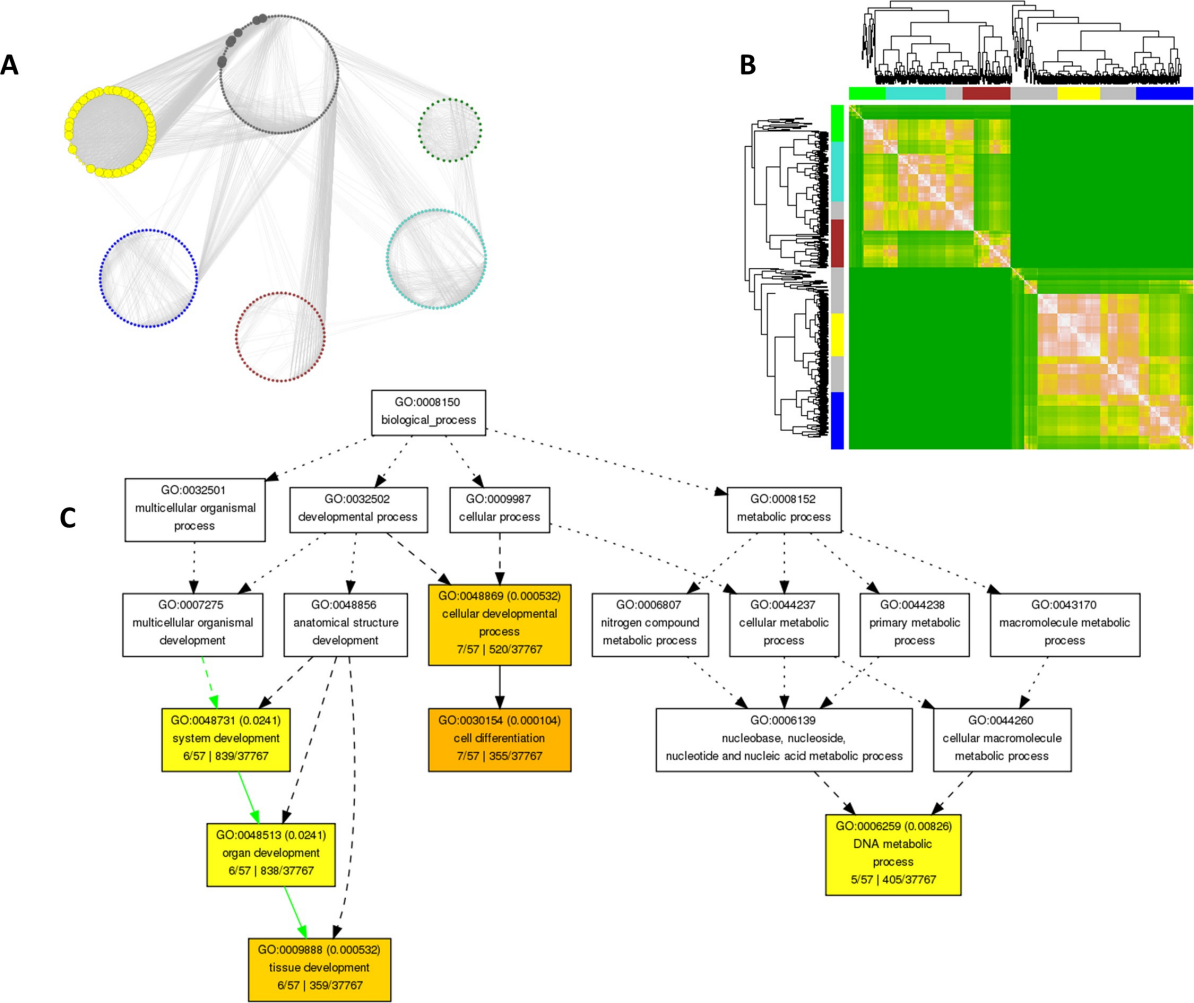
WGCNA for identification of hub genes among CLCuD-responsive DEGs in *G*. *hirsutum*. (A). TOM heatmap on 468 DEGs. Pink and green colors denote high and no strength, respectively, while dendrogram lines represent genes. DEGs with similar expression regulation patterns have been clustered and referred as modules. (B). Weighted correlations within the network deciphered six modules with a threshold of ≥ 0.85. The network nodes having more than 50 connections were denoted as highly connected hub genes (C). GO term annotation of CLCuD responsive hub genes revealed by WGCNA.

With the accessibility of large transcriptomic datasets, identification of a set of co-regulated genes under a stimulus or a physiological condition may be performed using co-expression network analysis [64, 65]. Thus, a co-expression network may help identify a cohort of genes that are involved in a shared biological process. Towards this, we performed a WGCNA to reveal diverse co-regulated gene groups in our RNA-Seq dataset [33, 34]. Gene expression similarity matrix between two nodes was computed by neighborhood proximity and depicted by creating topological overlap mapping metric (TOM) plot [34]. TOM also made a display of dendrograms with weighted correlations [66]. The overall analysis generated an undirected co-expression network possessing six different modules that are demonstrated in six different colors **(Fig 7A and 7B and S3 Table)**. Specifically, these modules *i*.*e*. grey, turquoise, blue, brown, yellow and green consist of 136, 81, 78, 65, 58 and 51 genes, respectively. Most significant connections or nodes were deciphered within the weighted co-expression network by making degree and clustering coefficient analysis [67, 68]. The network topological property analysis discovered overall 55 hub genes with ≥ 50 connections in the co-expression network (**Figs 3B and 7B and S4 and S5 Tables**).

### CLCuD-related modules provide insight into the establishment of disease susceptibility in cotton

Intriguingly, all of the hub genes, discovered in co-expression network are downregulated and are enriched in yellow module. GO term annotation of these hubs shown their association with intracellular processes (**Figs 7C snd S2**). Like all other viruses, geminiviruses have coevolved with their host plants. These viruses overcome the plant defenses and hijack the endogenous host cellular processes for their replication, expression and transport of their DNA genomes. Therefore, hub genes that include transcription factors, kinases, phytohormones, DNA repair, plasmodesmata and organelle trafficking-related genes possibly play roles in successful establishment of viral infection leading to heightened susceptibility to CLCuD in cotton. Gene families of particular interest as potential targets for engineering pathogen resistance are further elaborated below.

#### Transcription factors

Transcription factors play as major regulators to govern the expression of multiple genes under different biotic and abiotic stresses in plants. In particular, they have been focus of many studies involved in disease resistance mechanisms in several plants species for their regulatory actions in activating defense-responsive genes [69]. In our analysis, several transcription factors are differentially expressed in cotton under CLCuD infection including zinc finger, MYB, WD-40 repeat family proteins, NAC domain and bHLH. These results are in concert with a previous study that displays the differential regulation of these transcriptional gene families in cotton in response to whitefly infection [26]. In addition, the roles of these transcription factors have also been studied to be associated with viral infections in plants. Host NAC transcription factor directly interacts with the geminiviral proteins upon disease infection and this interaction leads to enhanced viral replication [70]. Consistent with these findings, downregulation of NAC transcription factor in our data might indicate its role in establishment of early disease infection. Moreover, MYB transcription factors regulates an array of genes involved in diverse plant processes including development, hormone signaling, metabolism, plant stress and disease resistance [71]. In another study, the association of bHLH transcription factors and *Tomato yellow leaf curl virus* disease resistance in tomato (*Solanum lycopersicum*) [72] has been demonstrated. In our experiment, all these transcription factors were downregulated suggesting their roles in suppression of cotton defense-responsive genes that are implicated in disease resistance against whitefly and CLCuD.

#### Phytohormone signaling

Differential gene expression in phytohormones in plants upon pathogen infection has been well studied [73]. CLCuD infection in *G*. *hirsutum* revealed the DEGs associated with phytohormone signaling pathways. In this study, auxin- and cytokinin-related genes were upregulated that highlight their roles in plant defense responses under this deadly viral attack. Allene oxide synthase (AOS) gene, which encodes an essential enzyme in jasmonic acid (JA) biosynthesis, was also downregulated in our data. Previously, the AOS transcript and cellular concentration of JA were found to be critical for pathogen and viral infection in plants [74]. Hence, downregulation of AOS in our experiment suggests its role in the downregulation of JA pathway leading to the cotton susceptibility to the disease. Abscisic acid (ABA) is another important plant hormone that functions as a chemical signal in response to different biotic and abiotic stresses. ABA further triggers an array of genes involved in physiological and developmental processes of the plant to cope with the stress conditions [75]. If activated at early stages of the disease, ABA is involved in the positive regulation of plant defense against invader pathogens but in case of later stages of infection, ABA suppresses reactive oxygen species production, JA and salicylic acid pathways, thus negating the plant defenses by manipulating these hormonal signaling cascades [76]. This suggests that ABA is involved in both pre- and post-invasive plant defenses. ABA limits the movement of viruses by increasing callose deposition and is shown to be required for disease resistance against diverse viruses including TMV and PVX [77]. Likewise, another study demonstrates that tomato plants resistant to TMV contained elevated levels of ABA compared to susceptible tomato [78]. Similarly, ABA is involved in the regulation of miRNA and siRNA pathways including dcl1-11, HUA ENHANCER 1 (hen1), dcl2, dcl3 and dcl4, which are negative regulators of geminivirus infection in host plants [79, 80]. In concordance with these observations, downregulation of genes that are involved in ABA pathways in our data suggests a role of ABA in limiting the induction of imperative genes involved in mRNA processing, miRNA/siRNA biogenesis and other hormones, thereby compromising the plant defense against whitefly and CLCuD. Taken together, we concluded that phytohormone-related complex gene expression under whitefly-mediated CLCuD shows the important implication of these signaling pathways in cotton as response to CLCuD infection.

#### Protein kinases

Protein kinases play an essential role in plant-pathogen interactions and plant immunity. Kinase gene family has also been found to be involved in plant metabolism and other cellular processes [81]. These can also concert with the pathogen proteins to facilitate pathogen for infection particularly in case of viral infections. Host protein kinases have been reported to implicate such processes by interacting with geminivirus proteins [82]. In our experiment, we found NAD kinase 2, SIK1, leucine-rich repeat transmembrane protein kinase, ACT-like protein tyrosine kinase related genes were downregulated upon infection of CLCuD, while thiamin pyrophosphokinase1 and a CBL-interacting protein kinase remained upregulated. The downregulation of most of the kinases in CLCuD plants might indicate their role in supporting the viral infection, while upregulation of some of these could possibly maintain plant metabolism, aiding plant survival under viral propagation.

#### Role of metabolism in response to CLCuD

Upon pathogen infection, energy requirements of the plants are increased under a pathogen stress. These requirements are then supported by involvement of primary and secondary metabolic pathways. Secondary metabolites comprising lignin and phenolics are requisite for plant for coping safeguard against microbial pathogens [83]. We identified two modules grey and turquoise that are enriched with genes-related to cellular metabolism (**S3 and S4 Figs**). These genes include flavonoids, aromatic compounds metabolism and galactose metabolism-related genes. S-adenosyl-L-methionine-dependent methyltransferases, cytochrome p450, HSP chaperones were upregulated, while galactose metabolism-related genes including alpha-galactosidase 1 and UDP-glucose pyrophosphorylase 2 were downregulated. All these genes are well studied to have a role in plant defense responses against viral and insect pathogens [84–86]. Previous studies have shown accumulation of flavonoids in cotton under sap sucking insects and fungal pathogen infections and revealed that flavonoids act as signal molecules for defense response mechanisms. Also sugar metabolism-related genes have been found to be differentially expressed under aphid infestation in plants [87, 88]. The differential gene expression of these sets of genes in CLCuD infected plants might indicate their potential role in coordinated plant metabolism under stress conditions.

### Differential gene expression of methyltransferases and protein modifications-related genes in response to CLCuD

We found three modules *i*.*e*. blue, brown and green that are enriched in genes involved in immune response and intracellular processes (**S5–S7 Figs**). Host plants use DNA methylation as an innate immune signal to regulate diverse array of downstream genes and suppresses endogenous transposons and invading DNA viruses. Geminiviruses encoding proteins inhibit host methylation and therefore represses transcriptional gene silencing (TGS) as a counter-defense. TrAP/C2 of geminivirus suppresses host TGS to establish viral spread in the host plant [89]. In our data set, the downregulation of DNA methylation-related gene SUVH3 suggests its role in the repression of host TGS to stabilize viral infection. Post-translational modification of proteins is necessary in the defense response to a pathogen infection. Pathogens modify post translational modifications machinery of host to trigger susceptibility in host [90]. Therefore, we found protein processing and ribosome-related differentially expressed genes in our data that propose roles of these genes in aiding pathogen to enhance disease susceptibly in cotton.

Previous transcriptomic study between cotton and its pathogens were performed on different regimes or genotypes. For instance, gene expression analysis of highly resistant and susceptible cotton varieties upon infection with aviruliferous whitefly showed the role of MPKs, WRKY factors, JA, ET and metabolism-related genes in insect resistance [27]. Additionally, cationic peroxidase 3, lipoxygenase I, TGA2, non-specific lipase, amino acids biosynthesis and carbon fixation related genes have been found influenced in response to aphids and whiteflies infestation [26]. Moreover, RNA-Seq dataset on diploid cotton *G*. *arboreum* under graft-mediated CLCuD infestation indicated the involvement of Aquaporin TIP4-1, NRT1/PTR and SWEET transporters in long-sought transport of secondary metabolites and defense-related compounds as a defense response strategy [47].

However, in our transcriptomic study on a susceptible cultivar karishma cotton under whitefly-mediated CLCuD infestation, we identified a different set of differentially expressed genes including NAC, MYB, bHLH, alpha-galactosidase, methyltransferases, cytochrome p450 and HSP chaperones. Intriguingly, most of identified hub genes are downregulated and therefore, the under-expression of such highly co-expressed genes suggests their roles in favoring the whitefly and virus and enhancing plant susceptibility to CLCuD. We have also discussed the role of abscisic acid, transcriptional gene silencing and post translational modifications in compromising the plant defense against whitefly and CLCuD. We have discovered differentially expressed genes in tetraploid susceptible cotton karishma variety under field like stress conditions of whitefly-mediated CLCuD infestation. Hence, these identified genes are different from genes identified in other studies. It is concluded that the variations in differential gene expression under different experiments is quite possible, as these variations come from different varieties, genotypes, ploidy levels of plants and/or stress conditions.

## Conclusion

In this RNA-Seq based study, we have shed light on the indispensable understanding of response of *G*. *hirsutum* to whitefly-mediated CLCuD infection. *G*. *hirsutum* is naturally susceptible to CLCuD and our study has revealed a complicated gene network involved in interaction of whitefly-transmitted CLCuD in cotton. The transcriptomic data provided here is a valued source that provides an opportunity to further characterize CLCuD defense responsive gene network. Subsequently, this cotton transcriptomics study will benefit the researchers in better and deep understanding of the mechanisms involved in *G*. *hirsutum* susceptibility to CLCuD.

### Conflict of interest

The authors declare that the research was conducted in the absence of any commercial or financial relationships that could be construed as a potential conflict of interest.

## Supporting information

S1 Fig Quality assessment of trimmed FASTQ sequence data. (TIF)

S2 Fig GO term annotation associated with yellow module of co-expression network. (TIF)

S3 Fig GO term annotation associated with grey module of co-expression network. (TIF)

S4 Fig GO term annotation associated with turquoise module of co-expression network. (TIF)

S5 Fig GO term annotation associated with blue module of co-expression network. (TIF)

S6 Fig GO term annotation associated with brown module of co-expression network. (TIF)

S7 Fig GO term annotation associated with green module of co-expression network. (TIF)

S1 Table Overall differential gene expression in the transcriptomic data of *G*. *hirsutum* under CLCuD infection. (XLSX)

S2 Table Primers for validation of transcriptomic data using RT-qPCR. (XLSX)

S3 Table Weighted gene co-expression network analysis: Modules. (CSV)

S4 Table Weighted gene co-expression network analysis: Degree of modules. (CSV)

S5 Table Cluster coefficient analysis in the co-expression network. (CSV)
