# Supplementary figures and images for: Transcriptomic analysis of cultivated cotton Gossypium hirsutum provides insights into host responses upon whitefly-mediated transmission of cotton leaf curl disease
Source: PLoS One. 2019 Feb 7;14(2):e0210011. doi: 10.1371/journal.pone.0210011 (PMC6366760; doi:10.1371/journal.pone.0210011)

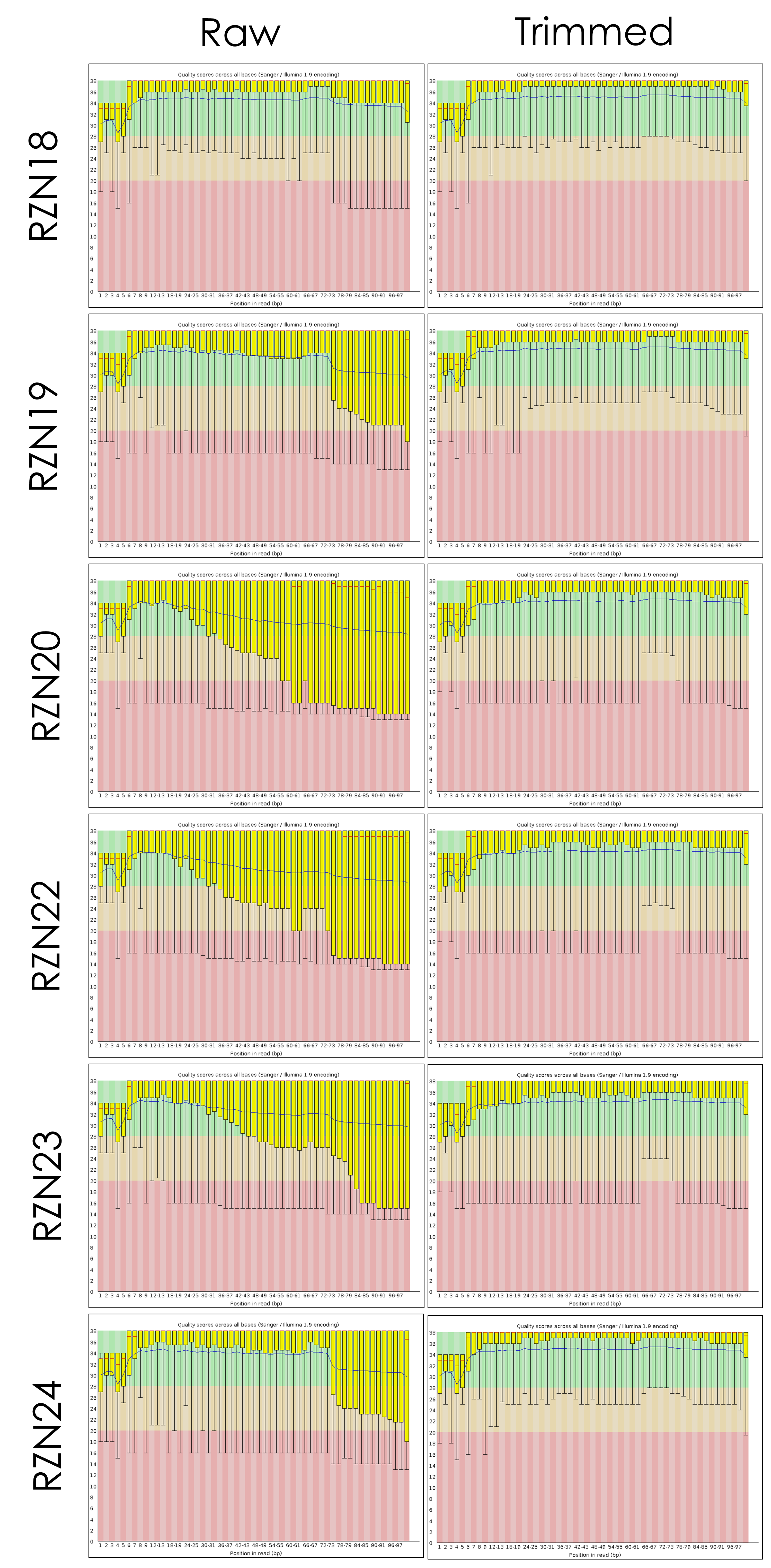

Supplement: S1 Fig — (TIF) [file pone.0210011.s001.tif]

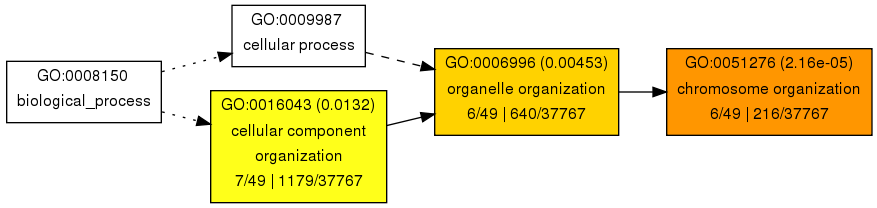

Supplement: S2 Fig — (TIF) [file pone.0210011.s002.tif]

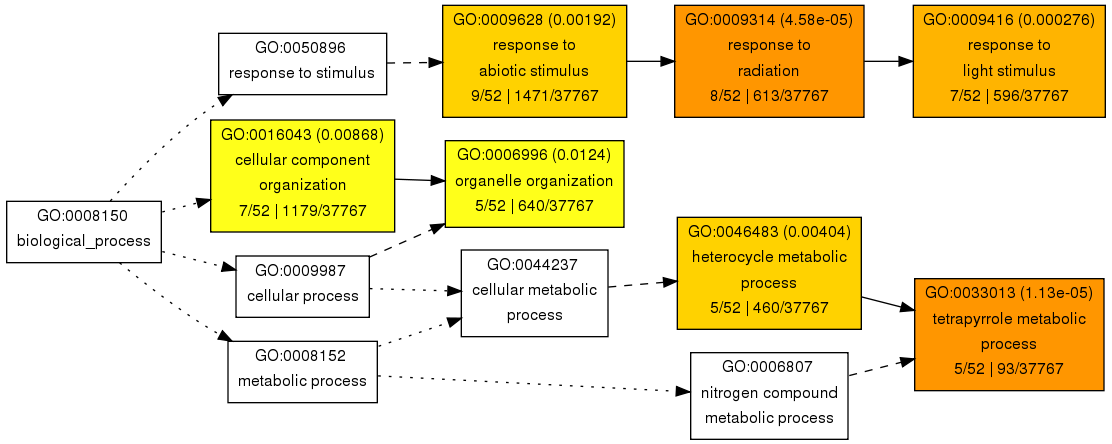

Supplement: S3 Fig — (TIF) [file pone.0210011.s003.tif]

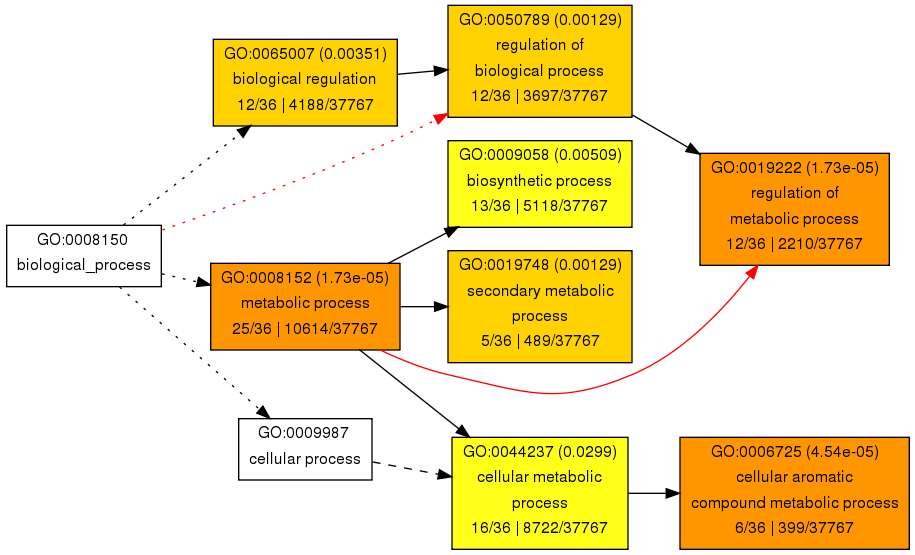

Supplement: S4 Fig — (TIF) [file pone.0210011.s004.tif]

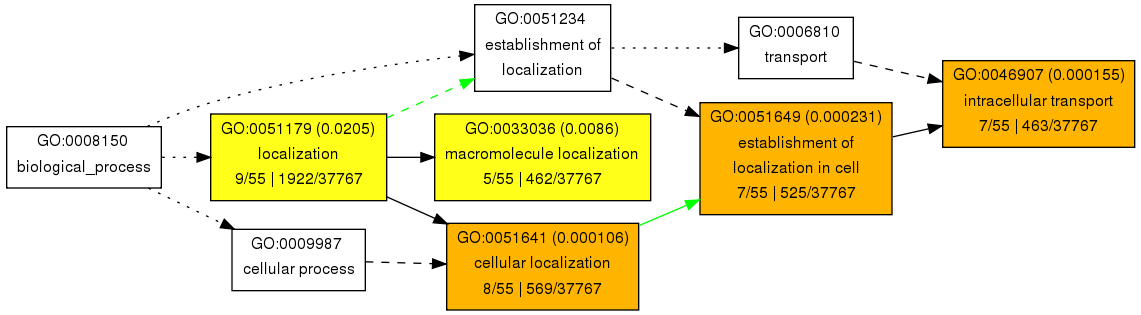

Supplement: S5 Fig — (TIF) [file pone.0210011.s005.tif]

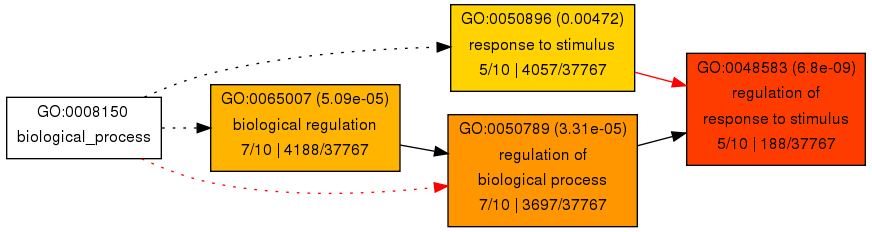

Supplement: S6 Fig — (TIF) [file pone.0210011.s006.tif]

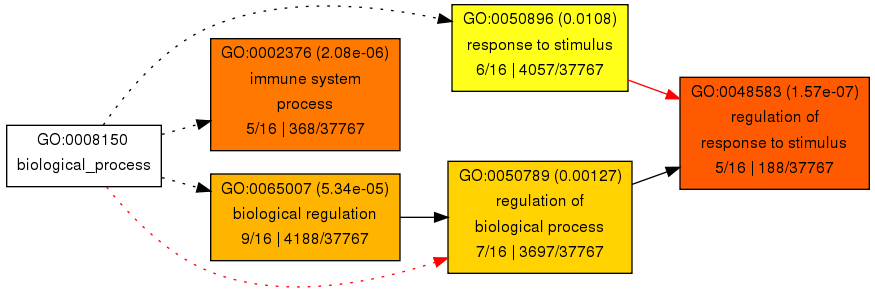

Supplement: S7 Fig — (TIF) [file pone.0210011.s007.tif]
